# Ehlers-Danlos Syndrome Type VIII: A Rare Cause of Leg Ulcers in Young Patients

**DOI:** 10.1155/2013/469505

**Published:** 2013-10-03

**Authors:** Sophie Ronceray, Juliette Miquel, Antoine Lucas, Gérald E. Piérard, Trinh Hermanns-Lê, Anne De Paepe, Alain Dupuy

**Affiliations:** ^1^Department of Dermatology, Pontchaillou University Hospital, 2 Rue Henri Le Guilloux, 35033 Rennes, France; ^2^Department of Vascular Surgery, Pontchaillou University Hospital, 2 Rue Henri Le Guilloux, 35033 Rennes, France; ^3^Department of Dermatopathology, Sart-Tilman University Hospital, B-4000 Liège, Belgium; ^4^Center for Medical Genetics, Ghent University Hospital, B-9000 Ghent, Belgium

## Abstract

Ehlers-Danlos syndrome type VIII (EDS-VIII) is a very rare autosomal dominant disease characterized by early-onset periodontitis associated with features of Ehlers-Danlos syndrome. We report a 32-year-old man whose chronic leg ulcer led to the diagnosis of EDS-VIII. He had severe periodontitis with complete loss of permanent teeth and skin fragility with thin skin, atrophic scars, and brownish atrophic pretibial plaques. Leg ulcer is not a prominent feature of EDS-VIII. We suggest adding EDS-VIII to the list of rare diseases accounting for chronic leg ulcers, if this case report prompts others to report leg ulcers associated with EDS-VIII.

## 1. Introduction

Ehlers-Danlos syndromes (EDS) are heritable connective tissue diseases caused by defects in the collagen meshwork. The main features include articular hypermobility, skin hyperextensibility, and connective tissue fragility. Ehlers-Danlos syndrome type VIII (EDS-VIII) is a very rare autosomal dominant disease with about 60 previously reported cases. Early-onset periodontitis with complete loss of adult dentition by the end of the third decade characterizes this entity [1]. Leg ulcer is not a prominent feature of EDS-VIII. 

We report the case of a 32-year-old man with recurrent leg ulcers leading to the diagnosis of EDS-VIII, and we discuss the opportunity to add EDS-VIII as a previously unrecognized cause of leg ulcer.

## 2. Case Report

A 32-year-old man was referred for clinical evaluation because of a persistent leg ulcer. He had suffered from leg ulcers for 15 years associated with superficial venous insufficiency treated with two phlebectomies. Family history revealed a complete loss of teeth at the age of 30 for his mother and a heterozygous alpha-1-antitrypsin deficiency in his paternal uncle and grandmother, with no known venous insufficiency. 

**Figure 1:**
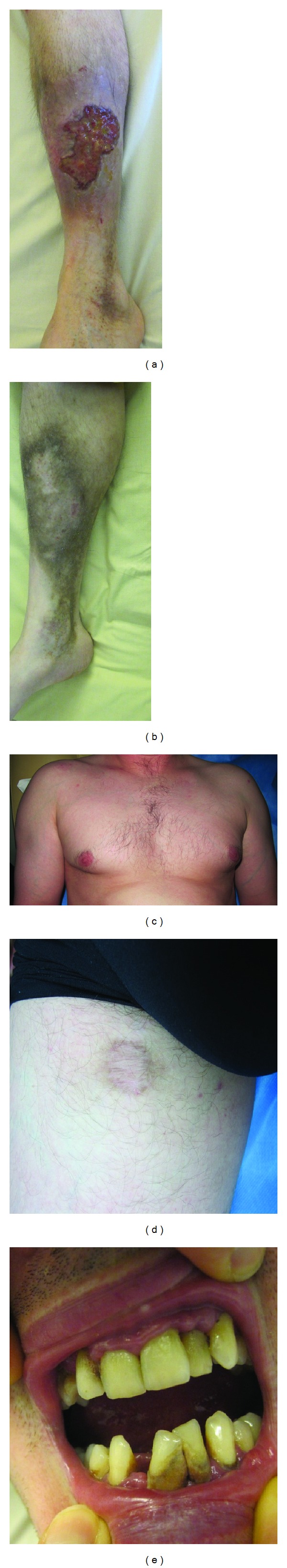
Typical features of EDS-VIII in our patient. (a) Leg ulcer within a brownish atrophic pretibial plaque. (b) Brownish atrophic plaque on the opposite leg. (c) Visible venous pattern on the trunk. (d) Atrophic scar on the right thigh. (e) Apical root resorption and gingival recession.

He weighed 79 kg for 1.73 m (BMI = 26 kg·m^−2^). He had a 6 by 9 cm ulcer on the anterior aspect of the left leg (Figure 1(a)) and bilateral brownish atrophic plaques on the pretibial areas mimicking necrobiosis lipoidica (Figure 1(b)). Distal pulses were present. His skin was thin with visible venous pattern on the trunk (Figure 1(c)). Four atrophic scars were noted on his right knee and both thighs (Figure 1(d)). There was no skin hyperextensibility or joint hypermobility. He had periodontitis with apical root resorption and gingival recession (Figure 1(e)). 

Laboratory studies disclosed normal values for total blood count, coagulation test, renal and liver function tests, serum protein electrophoresis, and hemoglobin electrophoresis. Antinuclear antibodies, antineutrophil cytoplasmic antibodies, antiphospholipid antibodies and cryoglobulinemia were negative. Karyotype and urinary amino acids chromatography were normal. Alpha-1-antitrypsin level was normal, and the patient had a homozygous wild-type genotype for alpha-1-antitrypsin gene. Aortic and lower limb doppler ultrasound ruled out an arterial disease and demonstrated varicose veins on the internal aspect of the left leg emerging from incontinent neosaphenous and perforating veins. Large arteries were normal on CT-scan. Dental X-ray showed generalized alveolar bone resorption. 

**Figure 2:**
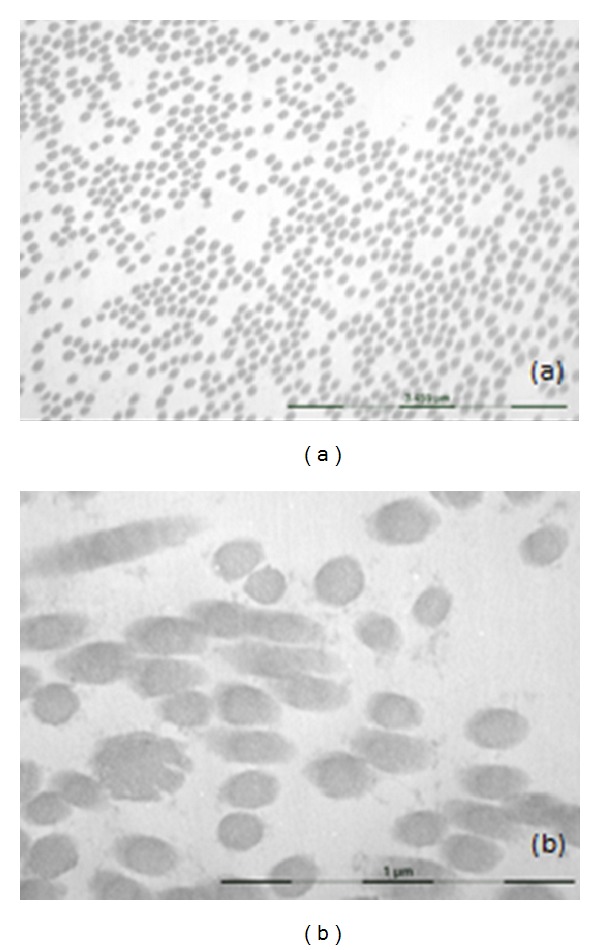
Transmission electron microscopy analysis on skin biopsy. (a) Irregular interfibrillar spaces (7750x). (b) Collagen cauliflowers (16700x).

The diagnosis of EDS-VIII was suspected on clinical grounds. Pathological examination of skin biopsies from the ulcer demonstrated pseudoepitheliomatous hyperplasia without malignancy. A biopsy from brownish atrophic periulceral skin demonstrated dermal fibrosis and hemosiderin deposition without images of necrobiosis lipoidica nor vasculitis. Transmission electron microscopy analysis on skin biopsy revealed irregular interfibrillar spaces (Figure 2(a)), collagen fibers with variable diameter, and collagen cauliflowers (Figure 2(b)). Electrophoretic migration pattern of the collagen type I, III, and V proteins from the patient's cultured fibroblasts was presented with a normal profile. 

During the year after his first visit, he had all his permanent teeth removed because of multiple dental mobilities and infections. The leg ulcer slowly healed thanks to daily care, type IV venous compression, and sclerotherapy of neosaphenous and perforating veins. 

## 3. Discussion

We describe a 32-year-old patient whose chronic leg ulcer led to the diagnosis of EDS-VIII. Our report provides the opportunity to add EDS-VIII in the list of rare diseases accounting for chronic leg ulcers. 

 The diagnosis of EDS-VIII was made on clinical grounds, since the molecular basis of this disorder is unknown. Characteristic clinical features in our patient included severe periodontitis and skin fragility with thin skin, atrophic scars and brownish atrophic pretibial plaques [2]. Other inconstant features of EDS-VIII, such as a marfanoid habitus, a characteristic triangular facies, minimal skin hyperextensibility, and moderate joint hypermobility, were not present. The same periodontal disease in his mother reinforced the hypothesis of a hereditary disease, and other causes of autosomal dominant periodontitis were ruled out [3, 4]. Morphological abnormalities of collagen were observed in our patient's skin. These were previously described in EDS-VIII as nonspecific collagen alterations, especially variations in collagen fibril diameter [3–6]. A reduction of synthesis of type I and III collagen was reported in the skin of two EDS-VIII patients [6, 7]. However, other reports failed to demonstrate any abnormalities in type I or III collagen [2, 3, 5, 8]. For all the above reasons, we are confident in the diagnosis of EDS-VIII in our patient.

 One peculiar feature of EDS-VIII is the necrobiosis lipoidica-like brownish plaques on the anterior aspects of the legs. Although they may not be present in some families of EDS-VIII [9], they seem to be a pervasive feature since the first description in 1972 [1]. They are thought to be a result of extensive bruising and confluent atrophic scars because of the increased fragility of dermis and small blood vessels [4]. Interpretation of these plaques as simple stasis dermatitis [10] does not fully account for their clinical characteristics. Indeed, as in our patient, their clinical features are the brownish color, the atrophic aspect, the well-circumscribed border, and the atypical topography on pretibial areas [2–4, 6]. Pathological examination of skin biopsies from these lesions revealed nonspecific dermal fibrosis and hemosiderin deposition [2]. These plaques can be seen as a relatively peculiar feature of EDS-VIII. 

Chronic leg ulcers may occur within these atrophic pretibial plaques because of tissue fragility and delayed wound healing. Leg ulcer was not described as a feature in the largest EDS-VIII review [2]. However, among published cases, there is at least one other report of a chronic leg ulcer occurring within a pretibial plaque [10]. In our case, superficial venous insufficiency of the lower limbs was proven both clinically and on doppler ultrasound, but the leg ulcer was not typical of this etiology because of its topography within an atrophic pretibial plaque. Other causes of leg ulcers were ruled out, including rare conditions like Klinefelter syndrome, prolidase deficiency, and alpha-1-antitrypsin deficiency. 

Vascular manifestations of EDS are common especially in the vascular EDS subtype (EDS-IV) [11]. Patients with EDS-IV have a high risk for arterial fragility and rupture. Although several authors have emphasized a phenotypic overlap between EDS-IV and EDS-VIII [5, 8], arterial investigations were normal in our patient. Moreover, varicose veins are described in EDS especially in EDS-IV [12]. Despite skin fragility and vascular manifestations, no chronic leg ulcer is described in classical and vascular EDS subtypes. Only traumatic wounds with delayed healing have been reported. 

## 4. Conclusion

Clinical characteristics of EDS-VIII must be searched out in front of atypical leg ulcers especially in young patients. Discussing this diagnosis may lead to early detection of periodontitis in order to prevent the complete loss of teeth. Moreover, it is important to eliminate a vascular EDS subtype because of clinical similarities. In major dermatology textbooks, EDS-VIII is not listed among rare conditions that may present with chronic leg ulcers [13–17]. We propose to add EDS-VIII in the list of rare causes of chronic leg ulcers, if this case report prompts others to report leg ulcers associated to EDS-VIII.
